# Neural Activations Associated With Friction Stimulation on Touch-Screen Devices

**DOI:** 10.3389/fnbot.2019.00027

**Published:** 2019-05-29

**Authors:** Wanjoo Park, Muhammad Hassan Jamil, Mohamad Eid

**Affiliations:** Engineering Division, New York University Abu Dhabi, Abu Dhabi, United Arab Emirates

**Keywords:** active touch, haptic interfaces, neural signal processing, tactile display, EEG

## Abstract

Tactile sensation largely influences human perception, for instance when using a mobile device or a touch screen. Active touch, which involves tactile and proprioceptive sensing under the control of movement, is the dominant tactile exploration mechanism compared to passive touch (being touched). This paper investigates the role of friction stimulation objectively and quantitatively in active touch tasks, in a real human-computer interaction on a touch-screen device. In this study, 24 participants completed an active touch task involved stroking the virtual strings of a guitar on a touch-screen device while recording the electroencephalography (EEG) signal. Statistically significant differences in beta and gamma oscillations in the middle frontal and parietal areas at the late period of the active touch task are found. Furthermore, stronger beta event-related desynchronization (ERD) and rebound in the presence of friction stimulation in the contralateral parietal area are observed. However, in the ipsilateral parietal area, there is a difference in beta oscillation only at the late period of the motor task. As for implicit emotion communication, a significant increase in emotional responses for valence, arousal, dominance, and satisfaction is observed when the friction stimulation is applied. It is argued that the friction stimulation felt by the participants' fingertip in a touch-screen device further induces cognitive processing compared to the case when no friction stimulation is applied. This study provides objective and quantitative evidence that friction stimulation is able to affect the bottom-up sensation and cognitive processing.

## 1. Introduction

Touch-screen devices, such as mobile phones or tablets, have become increasingly more common in recent years. Touch-sensitive surfaces allow users to interact with the screen using a finger (single-touch, multi-touch, or whole-hand input) or a pen, thus extending the expressiveness of hand gestures. Even though the lack of physical keys enlarges the display screen, it also implies that all of the interactive components on the screen are inherently visual and thus rely on visual feedback. This may cause many interaction problems since users have to devote full visual attention to the interface, and can severely affect the performance of the user's primary task (e.g., walking) or secondary task (e.g., interacting with the device, Yatani and Truong, 2007, 2009). The high visual demand of touch-screen interfaces also raises an accessibility issue for people with visual impairments. Auditory feedback can provide an additional modality of interaction, however this type of feedback is not always ideal or appropriate (privacy of interaction, ambient noise, etc., Abidin et al., 2013).

Tactile feedback has been used as an additional media to reduce the visual demand (Hoggan et al., 2008). Previous studies have shown that tactile feedback can improve user performance on different tasks using touch-screen devices (Nishino et al., 2011; Kumazawa et al., 2016; Liu et al., 2018). The added-value of tactile feedback in touch-screen devices is typically evaluated using self-reporting (questionnaires or think aloud protocols) or on users' behavior during the interaction (task completion time, accuracy, error rate, etc.). While both methods have been used successfully for decades, they suffer from several limitations. Self-reporting can be inconsistent, unreliable, and difficult to reproduce [e.g., prone to be contaminated by ambiguities (Nisbett and Wilson, 1977), sometimes affected by social pressure (Picard, 1995), and difficult to gain real-time insights without disrupting the interaction]. On the other hand, metrics inferred from behavior do not provide information about users' mental states (e.g., a high reaction time can be caused either by a low concentration level or by a difficult task, Berka and Levendowski, 2007). Recently, it has been suggested that brain imaging techniques (such as electroencephalography, EEG) have the potential to address these limitations as it provides an accurate and precise measure of user mental states (Frey et al., 2016).

Touch interaction is classified as either passive or active. In passive touch, physical contact is controlled by an external party (environment or other human), such as when a friend taps us on the shoulder. On the other hand, active touch involves the active use of human body to explore the environment (such as when stroking a surface to learn about its texture). Interaction with a touch-screen device involves mainly active touch. A significant neuroscience research on touch has focused on the neuronal circuits that compare passive and active touch (Harsimrat Singh et al., 2014; Moungou et al., 2016). Studies found that active touch induces different neural mechanisms as compared to passive touch (Lederman, 1981; Kim et al., 1994; Simoes-Franklin et al., 2011; Moungou et al., 2016). For instance, tactile perception is found to be better with active touch (Hollins and Risner, 2000) whereas gating of sensory transmission (excitability of the primary somatosensory cortex to tactile input) is reduced with active touch (Cheron et al., 2000). Some studies suggested that somatosensory input generated during passive touch elicits responses of greater magnitude in the primary somatosensory cortex as compared to active touch (Kim et al., 1994; Blakemore et al., 1999). Another study utilized steady-state evoked potentials (SS-EPs) technique to compare brain responses to identical stimuli during active and passive touch (Moungou et al., 2016). Results revealed that friction modulation induced by active and passive exploration of a tactile display elicited significant SS-EPs, whereas the differences between active and passive touch SS-EPs were not significant. The neural mechanisms associated with the perception of physical properties, such as texture, stiffness or stickiness, is also well studied. Brain correlates of texture during both passive and active touch tasks is examined using fMRI (Simoes-Franklin et al., 2011) and EEG (Yeon et al., 2017).

Meanwhile, few studies are conducted to explore the neural mechanisms associated with active touch interaction with touch-screen devices. An early study demonstrated that cortical sensory processing in the contemporary brain is continuously shaped by the use of touch screen devices (Picard, 1995). In this study, the cortical potentials in response to mechanical touch on the thumb, index, and middle fingertips of touch screen phone users and nonusers is measured. The cortical potentials from the thumb and index fingertips were directly proportional to the intensity of use (estimated via built-in battery logs), suggesting that repetitive movements on the smooth touchscreen reshaped sensory processing from the hand and that the thumb representation was updated daily depending on its use. However, no tactile stimulation is incorporated in this study. Therefore, it is necessary to study objective and quantitative brain activities associated with friction stimulation during active touch interaction with a touch-screen device. Vardar et al. (2017) investigated how the parameters of tactile stimulation affects human perception of electrovibration on touch screens. Psychophysical responses and behavior data such as force and acceleration and measured and optimized parameters to maximize human perception are reported. However, studying neural mechanisms associated with friction stimulation during touch-screen interaction seems to be lacking in the literature. Although friction stimulation clearly affects the user experience, compared to the absence of friction stimulation, there is a lack of research on brain activation and cognitive processes associated with friction stimulation. The aim of the current study is to provide quantitative and objective data about the neural activations associated with friction stimulation as a user actively interacts with a touch-screen device.

In our previous work (Park and Eid, 2018), we introduced the first study to examine the role of tactile stimulation objectively and quantitatively in an active touch task representing natural interaction with a touch-screen device. Results demonstrated a significant difference in beta oscillation in the middle frontal area at the late period of the active touch task (650–1,000 ms) is measured. In this paper, we extend this study by increasing the number of participants in order to achieve statistically significant differences in cognitive processing associated with friction stimulation. Furthermore, due to potentially additional benefits in terms of affective response to tactile feedback (creating an enjoyable hedonic experience for the customer or affects the decision to purchase a product, Peck and Wiggins, 2006), the study explores the effects of friction stimulation on influencing emotional responses.

## 2. Materials and Methods

### 2.1. Participants

Twenty-six participants enrolled in this study (14 males age range, 20–39). All participants were right-handed (Edinburgh handedness inventory, 98.46 ± 10.37) and met all inclusion/exclusion criteria. The inclusion criteria were: an age range from 20 to 39, right-handedness with no previous knowledge about how to play guitar, and normal or corrected-to-normal vision and hearing. Exclusion criteria included persons with a history of neurological or psychiatric disorders, persons with an orthopedic problem in the right hand, and persons with more than 6 months learning experience in playing guitar. We used a virtual guitar for the active touch task, that is why those who play guitar were excluded from the experiment. The experimental procedure and participant recruitment were reviewed and approved by New York University Abu Dhabi Institutional Review Board (IRB #073-2017). Written informed consent was obtained from all participants. All research data were collected and analyzed under IRB guidance.

### 2.2. Experimental Setup

**Figure 1:**
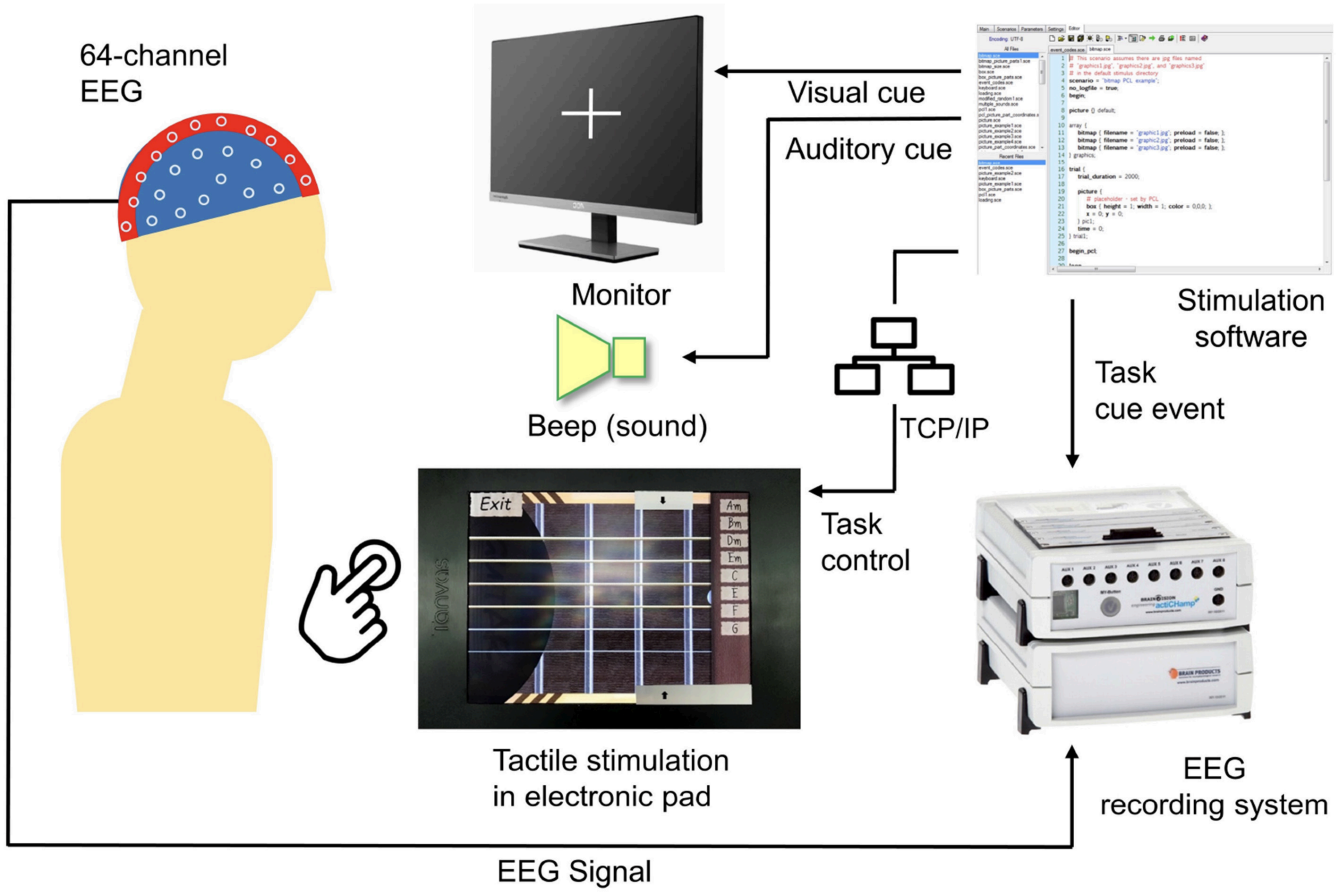
Block diagram of the experimental setup.

An active touch task was designed using a tactile touch-screen device while the EEG signal during the experiment was recorded. Figure 1 shows a block diagram of the experimental setup. The stimulation software was developed using Presentation (a software by Neurobehavioral Systems, Albany, CA, USA). This software controls visual and auditory cues and synchronizes these cues with friction stimulation displayed using the tactile touch-screen device, as well as records event trigger in EEG system.

To enable active touch task, a tactile touch-screen device developed by TanvasTouch^1^ was used to provide friction stimulation. This stimulation is achieved by modulating the surface friction between a fingertip and a physical display panel to simulate surface texture. Tactile touch-screen devices provide tactile feedback using electrostatic attraction of fingertip skin to a charged surface (Meyer et al., 2013, 2014).

Friction stimulation was activated programmatically via network socket communication between the virtual guitar application and Presentation software. The presentation control language (PCL by Neurobehavioral Systems) provides programmable access to Presentation software features including TCP/IP network interface to send and receive text messages from a network server. An initial network connection was established between Presentation software and the tactile touch-screen device via TCP/IP socket connection. After a successful connection, the Presentation software then controls the friction stimulation function on the touch-screen device through an activation message.

Neurological activities during an active touch task were recorded using a 64-channel EEG device and stored in the EEG recording system (BrainAmp by Brain Products, Munich, Germany).

### 2.3. Procedure and Evaluation Metrics

**Figure 2:**
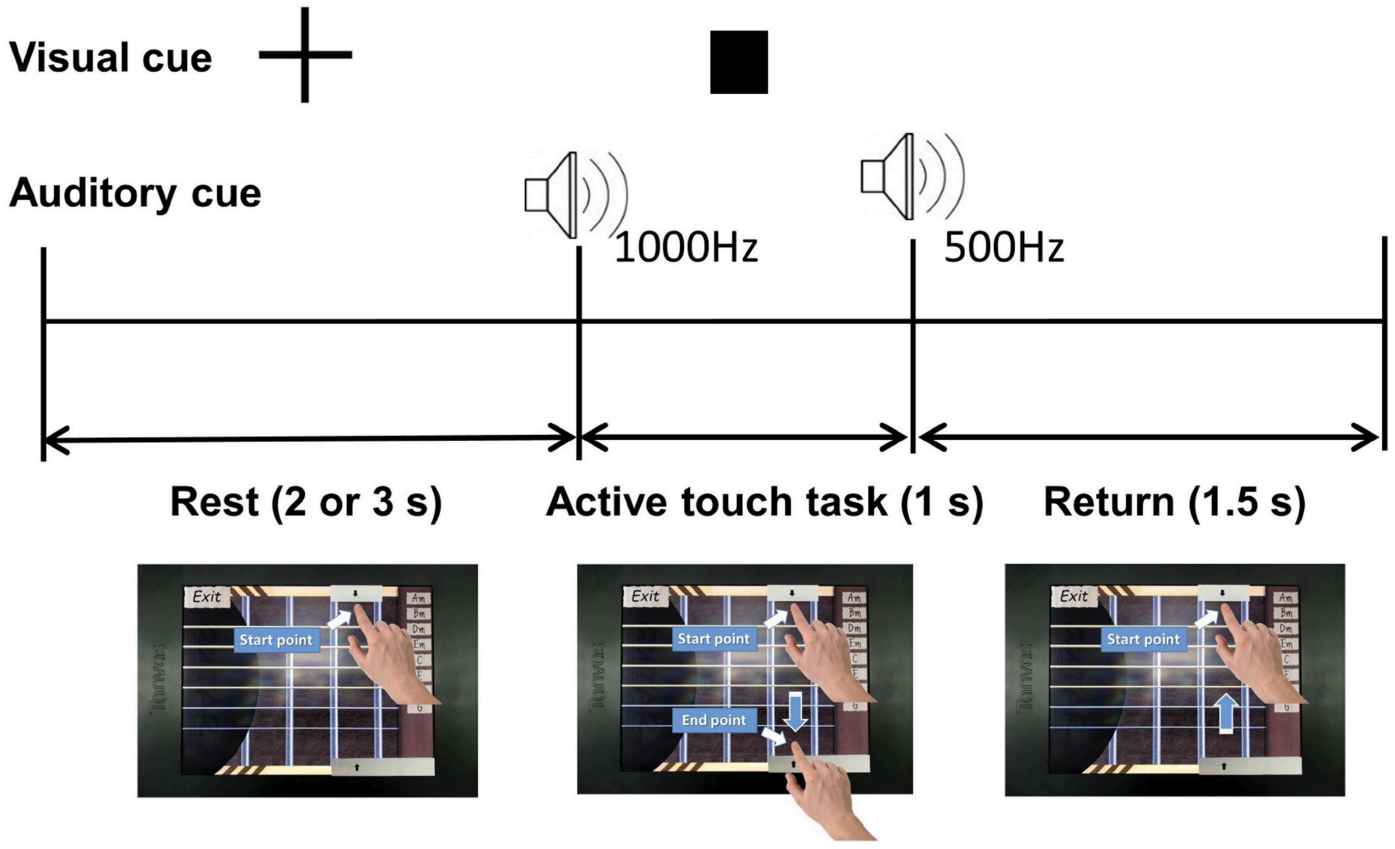
Schematic diagram of the experiment.

Participants were instructed to perform a task of stroking virtual guitar strings on the touch-screen device in the presence or absence of friction stimulation, at a random order. Figure 2 shows the schematic diagram of the experiment. One trial consisted of rest, active touch task, and return periods. The rest period was set to 2 or 3 s randomly to prevent participants from predicting task cues. The fixation appeared during the rest period to draw the user attention to the assigned task. The participant placed the index finger on the start point and waited for the active touch task as shown in the lower left in Figure 2. The square-shaped visual cue and a 1,000 Hz beep auditory cue announced the start of the active touch task. The participant moved their index finger from the start point to the end point within 1 s of the active touch task. At this time, the friction stimulation was enabled or disabled randomly to counter-balance the task order. However, the visual feedback (guitar strings are shaken) and audio feedback (string sound) are always provided while as the user's fingertip passed through the guitar strings. A beep sound of 500 Hz indicated the end of the active touch task. The return period was for 1.5 s, during that time participants moved their index finger back to the starting point while the rectangular visual cue disappeared. Before the experiment, all participants had a training session to reduce the variance of the finger movement time and minimize variations in their finger movement time beyond the allocated 1 s. In the experiment, one trial took 4.5–5.5 s and one run took 48 trials, therefore it took about 4.5 min for one run. All participants performed four runs and took three short breaks between successive runs. In total, we got 96 trial data for each presence/absence of friction stimulation per participant. EEG signals were recorded during all experiment.

Upon completing the experiment, participants were asked to complete a paper-based questionnaire about their experience. The participants rated valence, arousal, dominance, and satisfaction with five Likert scale in both cases; in the presence and absence of friction stimulation. Comparative analysis of EEG data is conducted between the two cases: with friction stimulation and without friction stimulation.

The EEGLAB toolbox was utilized for EEG signal processing (Delorme and Makeig, 2004). For preprocessing, EEG signals were down-sampled from 2,500 Hz to 1,250 Hz. Six EEG data streams corresponding to the outside locations (FT9, FT10, TP9, TP10, PO9, and PO10) were removed. A zero-phase finite impulse response filter was used for band pass filtering (0.1–55 Hz). A notch filter was applied with a zero-phase digital filter to remove the 50 Hz line noise. The artifact subspace reconstruction method was applied to remove eye movement and muscle artifacts (Mullen et al., 2013). Then, the filtered EEG signal was divided into epochs corresponding to when friction stimulation was applied or not. Finally, EEG signals were re-referenced using the common average reference (Binnie et al., 2003). After preprocessing, power spectral densities of theta (4–7 Hz), alpha (8–12 Hz), beta (13–30 Hz), and gamma (31–50 Hz) bands at each channel were computed via short-time Fourier transform. The differences associated with friction stimulation (when friction stimulation was applied or not) were analyzed through the topography of each frequency band in order to find areas of the brain that are most activated by friction stimulation. Spectrogram analysis was used to examine differences between theta, alpha, beta, and gamma bands for the with and without friction stimulation modes. Changes in power spectral density over time depending on the presence and absence of friction stimulation were also investigated. Event-related desynchronization (ERD) and synchronization (ERS) of alpha/beta bands for motor movement of participants were also examined in the contralateral motor and somatosensory areas (Pfurtscheller, 1997). The middle frontal/parietal area and the bilateral parietal area were selected as the regions of interest. In our previous study, friction feedback showed not only sensation but also gamma power difference in the middle frontal cortex (Park and Eid, 2018). Therefore, we expected significant differences in this region. We are also interested in the bilateral parietal area including the primary somatosensory cortex because the friction feedback is expected to play a role in the somatosensory area.

## 3. Results

**Figure 3:**
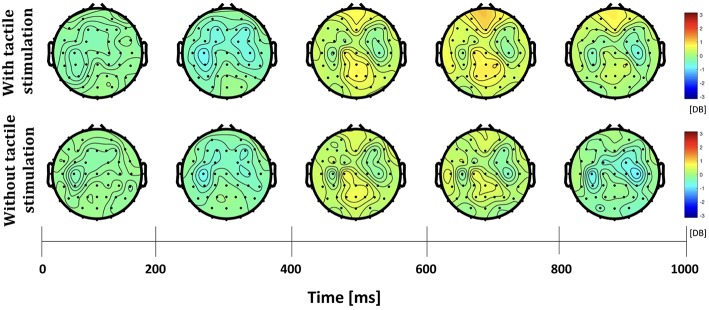
Topographies of beta power spectral density in the active touch task period.

**Figure 4:**
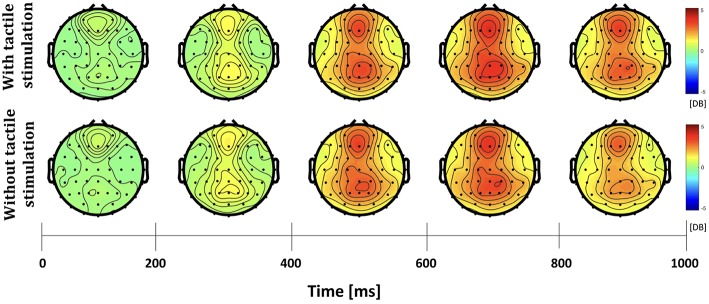
Topographies of gamma power spectral density in the active touch task period.

**Figure 5:**
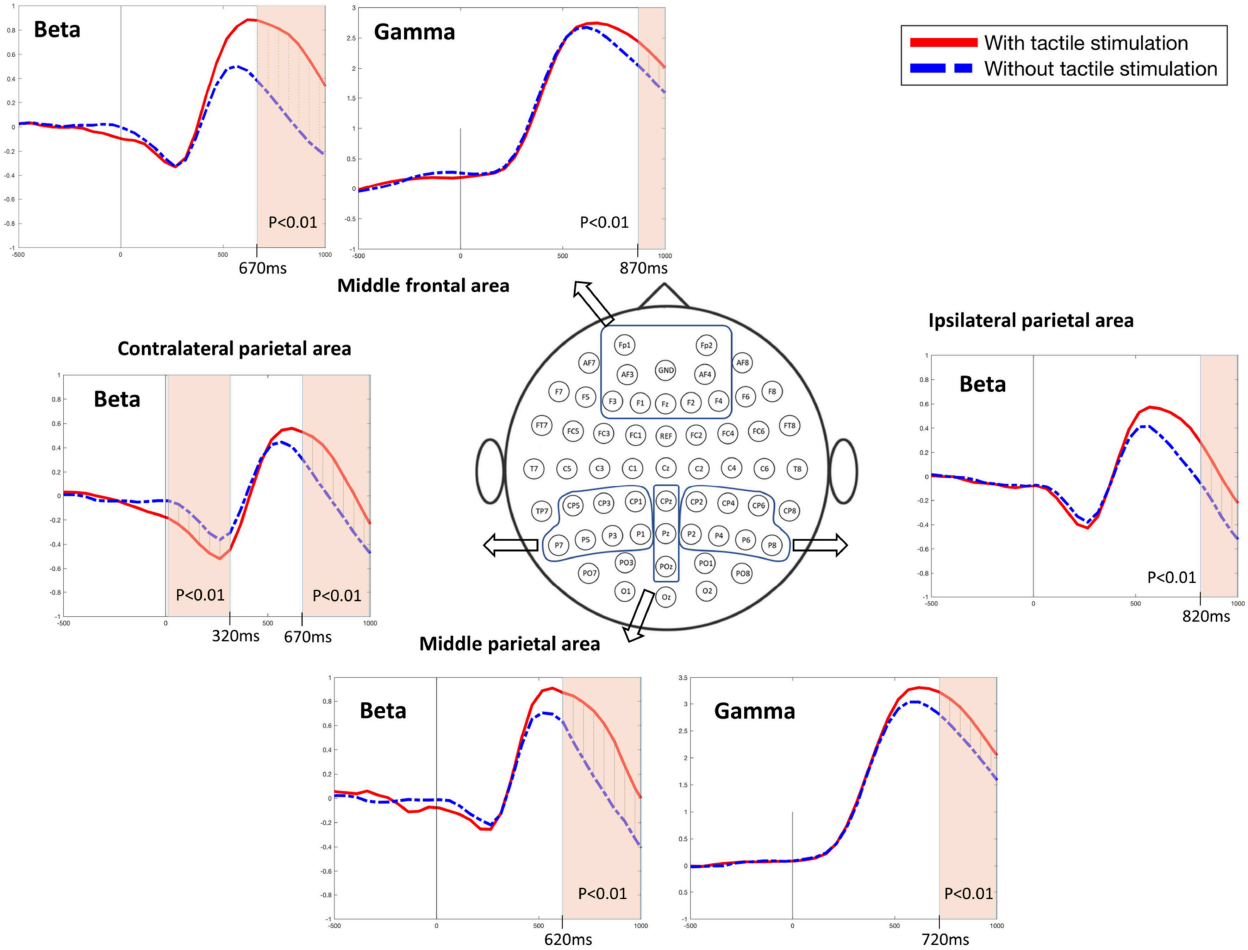
Beta and gamma power spectral densities in the middle frontal and parietal areas and beta power spectral densities in the bilateral parietal areas (*t*-test, *p* < 0.01).

The differences in frequency bands and brain regions associated with friction stimulation are examined. Figures 3, 4 show the cortical power distributions in beta and gamma oscillations. Significant differences of power spectral density (PSD) in beta and gamma oscillations in the middle frontal and parietal areas, as well as bilateral parietal areas, can easily be observed. Therefore, the analysis focused on four areas: the middle frontal area (Fp1, Fp2, AF3, AF4, F3, F1, Fz, F2, and F4), the middle parietal area (CPz, Pz, and POz), the contralateral parietal area (CP5, CP3, CP1, P7, P5, P3, and P1), and the ipsilateral parietal area (CP2, CP4, CP6, P2, P4, P6, and P8). Figure 5 shows the time course of PSD of beta and gamma oscillation in these four areas.

In the middle frontal and middle parietal area, beta and gamma PSDs when friction stimulation was applied were higher than in the case of no friction stimulation (*t*-test, *p* < 0.01). Beta PSD showed significant differences associated with friction stimulation after 670 ms, and gamma PSD showed a significant difference after 870 ms. However, in the middle parietal area, the differences associated with friction stimulation in beta and gamma PSDs were observed after 620 ms and 720 ms, respectively. These results indicate that differences in beta and gamma PSDs in the parietal area occurred 50 ms and 150 ms earlier than the middle frontal area, respectively.

In the contralateral parietal area, beta ERD was stronger in the early stage of a motor task (during 320 ms after motor task cue) when friction stimulation was applied, compared to the condition of no friction stimulation (Pfurtscheller and Da Silva, 1999). And then, beta rebound (Jurkiewicz et al., 2006) was also larger with friction stimulation and showed a significant difference after 670 ms (*t*-test, *p* < 0.01). We could not find a significantly different ERD in ipsilateral parietal area, but beta PSD associated with friction stimulation was higher only in the late period, i.e., after 820 ms (*t*-test, *p* < 0.01). Before the statistical tests, the normality of the sample data was confirmed by Jarque–Bera test and multiple compassion issues were also checked by Bonferroni correction.

## 4. Discussion

### 4.1. Middle Frontal and Middle Parietal Areas

Beta oscillations in the middle parietal and middle frontal areas have been reported to be highly related to emotional processing (McCabe et al., 2008; Harsimrat Singh et al., 2014; Lloyd et al., 2015; Marshall et al., 2015; Ravaja, 2017). For instance, hemisphere beta-oscillations distinguish most pleasant from least pleasant sensations (Harsimrat Singh et al., 2014). Top-down cognitive effects modulate the affective representation of touch and the sight of touch in the pregenual cingulate cortex and orbitofrontal cortex (McCabe et al., 2008).

**Figure 6:**
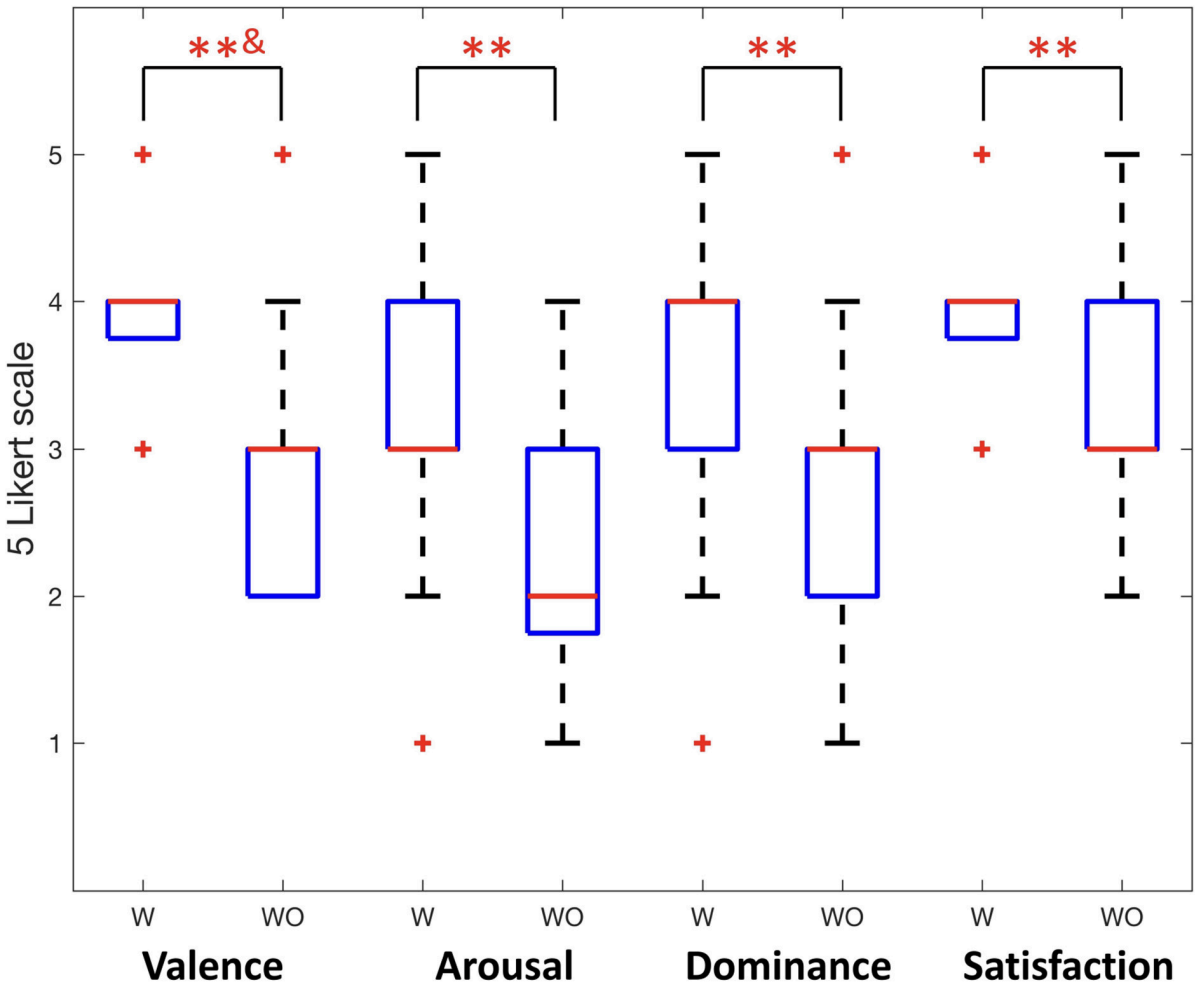
Participants' self reports for valence, arousal, dominance, and satisfaction (Wilcoxon signed rank test, ^**^&*p* < 0.01; paired sample *t*-test, ^**^p < 0.01). W indicates with friction stimulation and WO indicates without friction stimulation.

This was also confirmed in this study using subjective evaluation. Significant results are found in the questionnaire analysis. Figure 6 shows participants' self-rating with 5 Likert scale for valence, arousal, dominance, and satisfaction of their experience. When providing friction stimulation in all cases, we obtained a significantly higher score than otherwise (Wilcoxon signed rank test, *p* < 0.01; paired sample *t*-test, *p* < 0.01). Therefore, it was confirmed that not only the quantitative neural activity difference but also the subjective evaluation of the participants showed differences depending on the presence or absence of friction stimulation.

Furthermore, oscillatory activity within the beta-band provides distinct functional roles for attention and sensorimotor control, particularly for improved motor performance (Chung, 2017). This implies that friction stimulation plays a significant role in orient attention toward the interaction with the touch-screen device.

The dorsal cortical pathway is well known as one of the paths through which visual and auditory sensation is processed and transmitted. Neural signals from the visual and auditory cortex are combined in the parietal area and delivered to the frontal lobe (James and Kim, 2010). The differences in beta and gamma PSDs in the parietal area occurred 50 and 150 ms earlier than the middle frontal area, respectively. It can be interpreted in this context. However, neural activation of the ventral pathway can not be measured with EEG. Further neural connectivity study is needed to analyze the pathway of tactile sensation.

Gamma band oscillation is also well known to be related to cognitive processes, including attention, arousal, object recognition, and language perception (Herrmann et al., 2004). Previous studies have reported that sensory-cognitive dynamics and emotions are also correlated with gamma band oscillations (Başar, 2013) and object recognition and perception processing are also associated with gamma band oscillations (Başar et al., 2000; Herrmann et al., 2010). Furthermore, it has been observed in the literature that beta oscillations in the contralateral, middle, and the ipsilateral parietal areas are related to orienting attention (Marshall et al., 2015; van Ede et al., 2016). These findings are consistent with our findings.

### 4.2. Bilateral Parietal Areas

A recent study investigated the cortical responses induced by prolonged passive tactile stimulation and found long-latency bilateral and frontal somatosensory evoked potentials during passive interaction with a textured mechanical grating stimulus (Genna et al., 2017). The aforementioned study revealed significant ERD/ERS in the theta and alpha bands, respectively, in passive touch stimulation. The study revealed the increased power in the theta band started after the beginning of the passive stimulation that lasted for 500 ms followed by a decrease in alpha band power and lasted throughout the stimulation phase. In contrast, our study involves active touch stimulation of a fingertip on an electrostatic display touch screen device and revealed significant differences in beta and gamma bands oscillations in the bilateral parietal areas. ERD and rebound of beta PSDs were observed in all areas, regardless of whether friction stimulation is provided or not, as shown in Figure 5. This phenomenon has been observed in the perceptual processes (Panagiotaropoulos et al., 2013). The tactile touch-screen device provides audio and visual feedback, and even in absence of friction stimulation, it provides a sort of tactile feedback because the participants can feel the surface of the touch-screen device through the index finger. Thus, we suspect that this sensation triggered perceptual neural processing. However, at the late period of the active touch task, there were significant differences when friction stimulation is applied (*t*-test, *p* < 0.01). Beta PSDs associated with friction stimulation had risen more than without friction stimulation. It has been well know that beta oscillation reflects attentional, emotional and cognitive processes (Ray and Cole, 1985; Egner and Gruzelier, 2004).

An interesting observation is the time point of a significant difference in the presence or absence of friction stimulation. In the order of time, the first difference in beta ERD at the early period in the motor task was seen in the contralateral parietal area. Since all of the participants performed motor tasks with their right hand, it is reasonable to see a difference in the contralateral parietal area including the somatosensory cortex. In addition, there was a significant difference in beta rebound associated with friction stimulation from 670 ms, however, in case of the ipsilateral parietal area, this difference only appeared after 820 ms. It is well known that motor movement and tactile representation generally occur in the somatosensory motor cortex of the contralateral side, however the ipsilateral somatosensory motor cortex also plays a role in the motor task (Chen et al., 1997a,b). In addition, the contralateral motor cortex is activated first in the motor execution and then the bilateral motor cortex is activated and is responsible for the rest of the motor movement (Dimyan and Cohen, 2011). It is also consistent with this time order that these differences in the ipsilateral area appear later than the contralateral area.

## 5. Conclusion

This study provided objective and quantitative evidence associated with friction stimulation. This paper examined neural activations associated with friction stimulation as the user engages in active touch task with a touch-screen device. Results revealed significant differences in beta and gamma oscillations in the middle frontal and parietal areas with different time windows. In the contralateral parietal area, stronger beta ERD and rebound are observed when friction stimulation is applied, however difference in beta oscillation were only observed at the late period of the motor task in the ipsilateral parietal area. It is presumed that friction stimulation triggered the bottom-up sensation and cognitive processing, namely attention and control, which provides the user with better attention and control over their finger movements while interacting with a touch-screen device. Additional studies are needed to distinguish between bottom-up and top-down neural processing according to friction stimulation.

## Ethics Statement

The experimental procedure and participant recruitment were reviewed and approved by New York University Abu Dhabi Institutional Review Board (IRB #073-2017). Written informed consent was obtained from all participants. All research data were collected and analyzed under IRB guidance.

## Author Contributions

WP designed the experimental protocol and performed statistical analysis of recorded EEG data. MJ programmed and developed an application to perform and control tactile stimulation. WP and MJ managed hiring participants and conducting EEG experiments. ME supervised the study. All authors have contributed intellectually in writing and revising the manuscript.

### Conflict of Interest Statement

The authors declare that the research was conducted in the absence of any commercial or financial relationships that could be construed as a potential conflict of interest.

## Footnotes

^1^www.tanvas.co
